# Association of Coronoid Height Loss with Functional Outcomes in Terrible Triad Injuries Treated Using a Selective Fixation Algorithm

**DOI:** 10.3390/jcm15197624

**Published:** 2026-10-01

**Authors:** Yusuf Iyetin, Emre Koraman, Mehmet Akan, Ismail Turkmen, Muhlik Akyurek

**Affiliations:** 1 Department of Orthopaedics and Traumatology, Pendik Bolge Hospital, 34890 Istanbul, Turkey; y.iyetin@gmail.com; 2 Department of Orthopaedics and Traumatology, Kadikoy Florence Nightingale Hospital, 34724 Istanbul, Turkey; 3 Department of Orthopaedics and Traumatology, University of Health Sciences Sisli Hamidiye Etfal Hospital, 34371 Istanbul, Turkey; akanmehmet9@gmail.com; 4 Department of Orthopaedics and Traumatology, Klinikum Osnabruck, 49076 Osnabruck, Germany; dr.ismailturkmen@gmail.com; 5 Department of Orthopaedics and Traumatology, Maria-Josef Hospital, 48268 Greven, Germany; makyuerek@web.de

**Keywords:** terrible triad injury, coronoid height loss, elbow stability, selective fixation, radial head fracture, LUCL repair

## Abstract

**Background:** The purpose of this study was to assess whether coronoid fragment size, expressed as coronoid height loss, is associated with functional outcomes in terrible triad injuries treated using a selective coronoid fixation algorithm based on intraoperative elbow stability. **Methods:** This retrospective cohort study included adult patients with terrible triad injuries treated between 2016 and 2022. All patients underwent preoperative computed tomography evaluation with standardized measurement of coronoid height loss and fracture classification using Mason and Regan–Morrey/O’Driscoll systems. Radial head fixation or arthroplasty and lateral ulnar collateral ligament repair were routinely performed. Coronoid fixation was applied selectively, guided by intraoperative fluoroscopic stability assessment. Patients requiring secondary procedures for stiffness or radial head avascular necrosis were excluded from the final functional analysis, a potential source of attrition bias since these represent treatment failures rather than random missing observations. Functional outcomes at a minimum 24-month follow-up included elbow range of motion, Mayo Elbow Performance Score, and Quick Disabilities of the Arm, Shoulder and Hand score. **Results:** Functional outcomes were analyzed in 26 patients with coronoid fixation and 23 patients without fixation. Although coronoid height loss was higher in the fixation group, no significant differences were observed in range of motion or clinical outcome scores. Univariable and adjusted regression analyses did not demonstrate a significant association between coronoid height loss and range of motion or functional outcome scores. Findings were unchanged when coronoid fracture classification, determined independently of the continuous height-loss measurement, was used as the exposure variable. **Conclusions:** In this retrospective cohort, coronoid height loss was not significantly associated with functional outcomes in terrible triad injuries treated using a selective fixation algorithm. These findings suggest that intraoperative stability assessment may help guide coronoid fixation decisions; however, they should not be interpreted as proving that fragment size has no clinical relevance.

## 1. Introduction

The coronoid process is one of the primary static stabilizers of the ulnohumeral joint and plays a crucial role in preventing posterior translation and varus posteromedial rotatory instability of the elbow. Its biomechanical importance has been emphasized in numerous studies, which highlights how disruption of this structure can compromise elbow congruity and stability [1]. The terrible triad injury pattern—defined as posterior elbow dislocation combined with radial head fracture, coronoid process fracture, and lateral ulnar collateral ligament (LUCL) injury—represents a particularly complex and instability-prone scenario [2,3].

Complex injuries that include coronoid involvement, such as the terrible triad can lead to persistent subluxation, joint incongruity, mechanical dysfunction, and early ulnohumeral degeneration if not properly managed [1,4,5].

Despite the extensive biomechanical and clinical literature, the treatment algorithm for coronoid fractures—whether isolated or occurring as part of complex elbow instability—remains controversial [6]. While several classification systems exist, including the Regan–Morrey classification and the O’Driscoll classification, their ability to guide treatment decisively has been questioned. These systems may describe fracture morphology but do not reliably predict which coronoid fractures require surgical fixation [7].

Traditionally, fragment size has been considered a primary determinant of the need for fixation, most commonly expressed as the ≥50% coronoid height threshold derived from the Regan–Morrey classification. That threshold was established on lateral radiographs using a reference line drawn parallel to the ulnar shaft through the olecranon tip, and percentages obtained by different measurement conventions are therefore not numerically interchangeable. The recent literature has further challenged the notion that fragment size alone should dictate treatment [3]. Increasing evidence suggests that coronoid fragments, even when large, may not require fixation if elbow stability is restored through radial head reconstruction and LUCL repair. Conversely, seemingly small tip fractures can be relevant in specific instability patterns [3,8].

Several studies underline the importance of intraoperative stability assessment rather than fragment size as the primary determinant for fixation. According to recently published clinical evidence fluoroscopic evaluation of stability after radial head management and LUCL repair may be more predictive of functional outcomes than the absolute size of the fractured coronoid fragment [9,10]. Yet despite these insights, a definitive, universally accepted algorithm for determining when coronoid fixation is necessary in terrible triad injuries has not been established [3,9,10].

A notable gap in the existing literature is the limited number of clinical studies that assess the relationship between coronoid height loss, intraoperative elbow stability, and functional outcomes within a clearly defined and consistent treatment framework [1,3]. Previous research has emphasized the relevance of fragment size and the importance of intraoperative fluoroscopic assessment; however, most publications either combine heterogeneous injury patterns, do not quantify coronoid height loss using standardized computed tomography (CT)-based methods, or fail to examine how these morphologic variables interact with postoperative elbow function. As a result, uncertainty persists regarding the relative importance of fragment size versus intraoperative stability, particularly for Regan–Morrey type I–II and O’Driscoll tip or anteromedial facet fractures, where treatment recommendations remain variable across studies [1,3,9,10,11].

Accordingly, the aim of the present study was to evaluate the clinical and functional outcomes of terrible triad injuries managed using a selective fixation algorithm that incorporates both preoperative CT-based coronoid morphology and intraoperative dynamic stability assessment. Rather than proposing a new decision rule, this study sought to examine how this commonly used algorithm performs in a homogeneous cohort of terrible triad injuries and to determine whether coronoid height loss was associated with functional outcomes when intraoperative stability was restored.

It was hypothesized that, in Regan–Morrey type I–II and O’Driscoll tip or anteromedial-type fractures, coronoid height loss would not be significantly associated with functional outcomes once elbow stability was achieved intraoperatively following radial head reconstruction and LUCL repair.

## 2. Materials and Methods

### 2.1. Study Design and Patient Selection

This retrospective cohort study included adult patients (≥18 years) presenting with a classical terrible triad injury pattern—elbow dislocation associated with radial head and coronoid fractures—between January 2016 and December 2022. Institutional review board approval for retrospective review of this existing dataset was obtained prior to data extraction (Date: 30 April 2026, No: 2026/769). Written informed consent for participation in research and for the use of clinical and imaging data is obtained from all patients at admission as routine institutional practice. Patients were eligible if they had preoperative CT imaging, adhered to postoperative rehabilitation, returned for scheduled follow-up, and had a minimum of 24 months of clinical evaluation.

Patients with prior ipsilateral elbow surgery, pre-existing elbow arthritis or inflammatory arthropathy, systemic osteoporosis confirmed by DEXA, or concomitant ipsilateral upper extremity fractures were excluded. Patients requiring secondary surgical procedures (radial head excision for avascular necrosis (AVN) or manipulation under anesthesia for stiffness) were included in the cohort for demographic and fracture-type analyses but were excluded from final functional outcome assessment.

### 2.2. Imaging Protocol

All patients underwent thin-slice helical CT (General Electric Medical Systems Optima CT660 scanner (GE Healthcare, Milwaukee, WI, USA) with a 1-mm slice thickness) scanning of the elbow. Multiplanar reconstructions were reviewed in the sagittal, coronal, and axial planes. Fractures were classified according to the Mason classification for radial head fractures and the Regan–Morrey and O’Driscoll classifications for coronoid fractures. Fracture classification was based on fracture morphology and location on multiplanar CT reconstructions and was assigned independently of the continuous height-loss measurement.

Two fellowship-trained elbow surgeons (each with 10 years of experience in elbow surgery) independently evaluated all CT scans twice, with a 4-week interval between evaluations. Before formal assessment, both observers reviewed the predefined anatomical landmarks for coronoid height measurement and the fracture-classification criteria for the Mason, Regan–Morrey, and O’Driscoll systems to standardize the assessment protocol. Coronoid height measurements and fracture classifications were then performed independently by each observer. Interobserver and intraobserver reliability were calculated using intraclass correlation coefficients (ICC) and interpreted using established criteria: poor < 0.50, moderate 0.50–0.75, good 0.75–0.90, and excellent > 0.90. The ICC values for coronoid height measurements and for the Mason, Regan–Morrey, and O’Driscoll classifications were all >0.90, indicating excellent reproducibility. These reliability results were interpreted as evidence of measurement and classification consistency rather than proof of absolute diagnostic accuracy.

### 2.3. Coronoid Height-Loss Measurement

Coronoid height loss was measured on preoperative CT according to the method of Ahn et al. [3], in which height loss is expressed as the proportion of the total expected coronoid height represented by the fractured fragment. The centre of the proximal ulna was identified on the coronal reconstruction and used to select the corresponding mid-sagittal slice, which standardized the measurement plane and avoided oblique or eccentric assessment of the coronoid process. On this slice, two lines parallel to the ulnar shaft were drawn, one through the fracture surface of the residual coronoid process and one tangent to the base of the trochlear notch. The residual coronoid height (A) was measured as the perpendicular distance between these two lines, and the height of the displaced fragment (B) was measured on the fragment itself. The total expected coronoid height was taken as A + B, and coronoid height loss was calculated as B/(A + B) × 100 (Figure 1).

### 2.4. Treatment Protocol

Radial head fixation was performed first. Reconstructible fractures were managed with screw or plate-and-screw fixation, whereas comminuted Mason type III radial head fractures underwent uncemented radial head arthroplasty. Lateral ulnar collateral ligament repair was routinely performed.

Coronoid fixation was selective and based on mechanical criteria rather than morphology alone. The conventional 50% height-loss figure informed preoperative planning by prompting consideration of fixation, but it was not applied as a decision rule, and no numerical threshold determined whether fixation was performed. In every case the decision was made on the basis of the intraoperative stability test.

After these steps, stability was assessed under live lateral fluoroscopy with the forearm held in pronation. The elbow was taken passively from 90° of flexion to full extension under gravity, and varus and valgus stress was then applied at 30° of flexion. Persistent instability was defined as loss of concentric ulnohumeral reduction at any point during this assessment, identified on the lateral fluoroscopic view as widening of the ulnohumeral joint space or posterior translation of the ulna relative to the trochlea. Coronoid fixation was performed when such subluxation was demonstrated (Figure 2); if the elbow remained concentrically reduced throughout the tested arc and under stress, coronoid fixation was not performed. Implant selection and coronoid fixation technique were determined according to fracture morphology, fragment size, and bone quality; screw fixation, plate-and-screw constructs, or transosseous pull-out sutures were used accordingly. All procedures were performed or directly supervised by the same fellowship-trained elbow surgeon, and the decision for coronoid fixation was made according to the same staged intraoperative assessment protocol in all cases.

### 2.5. Rehabilitation Protocol

All patients were managed with a standardized postoperative rehabilitation protocol. After short-term postoperative immobilization, early protected motion was initiated using a hinged elbow brace. Active-assisted and active flexion–extension exercises and forearm pronation–supination exercises were performed within a stable arc according to postoperative elbow stability and soft-tissue condition. Varus stress, weight-bearing activities, resisted elbow exercises, and forced terminal extension were avoided during the early rehabilitation period. Progressive range-of-motion exercises were continued under supervision, and strengthening exercises were introduced at 8–10 weeks after surgery once stable painless motion had been achieved. The protocol included both supervised hospital-based rehabilitation and a structured home-exercise program. Written rehabilitation instructions and exercise checklists were provided to all patients. At scheduled follow-up visits, these checklists were reviewed, adherence to brace use and exercise progression was assessed clinically, and compliance with the prescribed rehabilitation program was documented in the medical records.

### 2.6. Clinical Outcome Measures

Final follow-up evaluation included measurements of flexion–extension arc, pronation–supination arc, the Mayo Elbow Performance Score (MEPS), and the Quick Disabilities of the Arm, Shoulder and Hand (QuickDASH) questionnaire. MEPS and QuickDASH evaluations were performed by an independent clinician blinded to coronoid fixation status. Complications were recorded systematically. Patients who required unplanned secondary surgical procedures were not included in the final continuous functional outcome analysis because postoperative range of motion and patient-reported scores could be influenced by the secondary intervention. However, these patients were retained in the overall cohort for baseline, fracture-type, complication, and secondary-procedure analyses.

### 2.7. Statistical Analysis

Normality was assessed using the Shapiro–Wilk test. Normally distributed data were analyzed with independent-samples t tests; non-normally distributed data with Mann–Whitney U tests. Continuous variables are reported as mean ± standard deviation together with median, interquartile range and range. Categorical variables were analyzed using chi-square or Fisher’s exact tests, as appropriate. Linear regression analyses were performed to evaluate the association between coronoid height loss and functional outcomes. Both univariable and adjusted models were constructed. In the adjusted models, coronoid height loss was entered as the predictor of interest, and age, sex, Mason type, radial head arthroplasty, and Regan–Morrey coronoid type were entered as covariates (except in the classification-based analysis described below, in which coronoid fracture type was the predictor of interest). Covariates were specified a priori rather than selected from the observed data: age and sex as patient-level determinants of elbow motion and patient-reported scores, Mason type and radial head arthroplasty as measures of radial head injury severity and its management, and Regan–Morrey coronoid type as a morphological descriptor independent of the continuous height-loss measurement. Given the sample size, no further variables were entered, and all covariates were available for every patient. For multiple functional outcome comparisons, *p*-values were additionally interpreted using the Holm–Bonferroni method. An all-treated sensitivity analysis was performed using unplanned secondary surgical procedure as an adverse endpoint, including all treated patients. All primary functional outcome comparisons were additionally repeated using coronoid fracture classification as the exposure variable in place of continuous coronoid height loss, in order to determine whether the findings were reproducible using an exposure variable independent of that measurement. A *p*-value < 0.05 was considered significant. The sample size calculation assumed a two-sided independent-samples t test with α = 0.05 and prespecified clinically relevant differences of 15° in the flexion–extension arc (SD 12) and 15 points in the MEPS (SD 14), corresponding to effect sizes of d = 1.21 and d = 1.07. A minimum of 16 patients per group was required, giving 91% and 83% power respectively; both analyzed groups exceeded this minimum. Statistical analyses were performed using IBM SPSS Statistics for Windows, version 28.0 (IBM Corp., Armonk, NY, USA), and the power analysis using G*Power version 3.1.9.7 (Heinrich Heine University, Düsseldorf, Germany).

## 3. Results

A total of 54 patients were included: 29 underwent coronoid fixation (Group 1) and 25 were treated without fixation (Group 2). Baseline demographic and fracture characteristics were analyzed in the overall treated cohort, including patients who required secondary procedures (Table 1 and Table 2). Five patients required unplanned secondary surgical procedures and were excluded only from the final continuous functional outcome analysis: three patients in Group 1 and two patients in Group 2. Therefore, final range of motion and patient-reported outcome analyses were performed in 26 patients in Group 1 and 23 patients in Group 2.

Groups were similar in age, sex distribution, and limb dominance (Table 1). The mean follow-up duration in the overall treated cohort was 28.11 ± 2.71 months, with a range of 24 to 33 months. Follow-up duration did not differ significantly between Group 1 and Group 2 (27.86 ± 2.85 vs. 28.40 ± 2.57 months, *p* = 0.40) (Table 1). The mean interval from injury to surgery in the overall cohort was 12.80 ± 4.16 h, with a range of 7 to 21 h. This interval did not differ significantly between Group 1 and Group 2 (13.34 ± 4.34 vs. 12.16 ± 3.92 h, *p* = 0.35) (Table 1). Mason type II and III fractures were evenly distributed across groups. Morrey and O’Driscoll subclasses also showed no significant difference (Table 2).

Coronoid height loss was numerically higher in Group 1 than in Group 2, but this difference did not reach statistical significance (50.21 ± 1.89% vs. 49.04 ± 2.74%, *p* = 0.09; Table 2). In Group 1, coronoid fixation followed demonstration of persistent ulnohumeral subluxation on the intraoperative stability test; no patient underwent fixation on the basis of fragment size alone.

Flexion–extension arc, pronation–supination arc, MEPS, and QuickDASH scores did not differ significantly between the fixation and non-fixation groups. After Holm–Bonferroni correction for multiple functional outcome comparisons, all between-group comparisons remained non-significant, with adjusted *p*-values of 1.00 for flexion–extension arc, pronation–supination arc, and MEPS, and 0.55 for QuickDASH (Table 3). In univariable linear regression analysis, coronoid height loss was not significantly associated with flexion–extension arc (β = –0.16, 95% CI: –0.92 to 0.61, *p* = 0.68), pronation–supination arc (β = –0.87, 95% CI: –2.76 to 1.02, *p* = 0.36), MEPS (β = 0.14, 95% CI: –0.73 to 1.01, *p* = 0.75), or QuickDASH (β = –0.31, 95% CI: –0.96 to 0.34, *p* = 0.34). In adjusted regression models, coronoid height loss remained non-significant for flexion–extension arc (β = –0.16, 95% CI: –0.99 to 0.67, *p* = 0.70), pronation–supination arc (β = –0.80, 95% CI: –2.69 to 1.09, *p* = 0.40), MEPS (β = 0.12, 95% CI: –0.81 to 1.06, *p* = 0.79), and QuickDASH (β = –0.36, 95% CI: –1.04 to 0.32, *p* = 0.29) (Table 4). 

### 3.1. Analysis Using Coronoid Fracture Classification

Regan–Morrey and O’Driscoll classifications were fully concordant across the cohort, with all Regan–Morrey type I fractures corresponding to O’Driscoll type 1 (tip) fractures and all type II fractures to O’Driscoll type 2 (anteromedial facet) fractures (n = 49). A single classification variable was therefore used for this analysis.

No significant differences in functional outcomes were identified between coronoid fracture types (Table 5). The flexion–extension arc was 125.1 ± 12.6° in type I and 125.7 ± 12.2° in type II fractures (*p* = 0.88), the pronation–supination arc was 145.1 ± 29.9° and 131.2 ± 30.4° (*p* = 0.10), MEPS was 86.7 ± 13.5 and 84.6 ± 14.5 (*p* = 0.79), and QuickDASH was 22.9 ± 10.4 and 26.1 ± 10.6 (*p* = 0.20), respectively. Univariable and adjusted regression analyses showed no significant association between coronoid fracture type and any functional outcome, and all Holm-adjusted *p*-values exceeded 0.05.

### 3.2. All-Treated Sensitivity Analysis, Secondary Procedures, and Complications

The all-treated sensitivity analysis included the overall cohort of 54 patients and used unplanned secondary surgery as a separate adverse endpoint rather than as a continuous functional outcome. Five patients required unplanned secondary surgical procedures. Group 1 experienced three such events: two cases of postoperative stiffness, one requiring implant removal with manipulation under anesthesia and one requiring manipulation under anesthesia alone, and one case of radial head AVN requiring radial head excision. Group 2 experienced two events, both due to radial head AVN requiring radial head excision. The secondary-procedure rate was 10.3% in Group 1 and 8.0% in Group 2, with no statistically significant difference between groups (*p* = 1.00). The five patients who required unplanned secondary procedures did not differ substantially from the remaining patients in baseline or fracture characteristics. Their mean age was 44.2 ± 8.6 years (four men, one woman), and mean coronoid height loss was 49.8 ± 2.3%. Two had Regan–Morrey type I and three type II coronoid fractures, and two had Mason type II and three Mason type III radial head fractures. No patient in this subgroup had a height-loss value, fracture type, or demographic characteristic outside the range observed in the remaining cohort. Because this analysis addressed a different endpoint, it does not compensate for the exclusion of these five patients from the continuous functional outcome analysis. No redislocation, persistent instability, infection, or nerve palsy occurred.

## 4. Discussion

The principal finding of this study is that, in terrible triad injuries managed with a selective fixation algorithm, coronoid height loss was not significantly associated with postoperative range of motion or functional outcome scores after intraoperative restoration of elbow stability. Comparable outcomes were obtained across the cohort in Regan–Morrey type I–II and O’Driscoll tip or anteromedial facet fractures managed under this algorithm. In the adjusted analyses performed in this cohort, coronoid height loss remained non-significantly associated with postoperative range of motion and functional outcome scores. These findings should not be interpreted as demonstrating equivalence between fixation and non-fixation or as proving that fragment size has no clinical relevance. Because allocation to fixation depended on the intraoperative stability test rather than on randomization, these findings describe the outcomes achieved under this algorithm rather than the independent prognostic value of fragment size.

Terrible triad injuries represent a highly unstable pattern involving posterior dislocation, radial head fracture, and coronoid fracture, affecting both primary and secondary stabilizers of the elbow, with residual instability and stiffness being common complications if stability is not restored appropriately [6,12]. Achieving a concentric ulnohumeral reduction and stable joint alignment is considered essential to allow early motion and prevent instability and stiffness [12]. Although the coronoid is an important bony stabilizer, traditional treatment approaches relied heavily on fragment size—particularly the conventional >50% height rule—as the main reason for fixation. However, many recent studies have questioned the reliability of size-based thresholds and have emphasized the importance of fracture type, associated soft-tissue injuries, and the ability to restore stability once the radial head and lateral ligament complex are repaired [12].

Biomechanical and clinical data have shown that the coronoid’s contribution to stability depends on the overall injury pattern. When the radial head is reconstructed and the LUCL is repaired, stability can often be restored even in the presence of tip or small anteromedial facet fractures [8]. Clinical reports have similarly demonstrated excellent outcomes in terrible triad injuries when Regan–Morrey type I–II fractures were left unfixed, provided that the radial head and lateral structures were adequately treated [7]. Studies focusing on coronoid tip fractures in terrible triad injuries have also shown no functional advantage for routine fixation, and have reported comparable range of motion in patients treated with or without fixation [10]. These findings are consistent with the present results, where functional outcomes were comparable despite slightly higher height-loss values in the fixation group.

Studies on isolated coronoid fractures have also shown that fragment size alone does not predict outcome. Nonsurgical management has produced good results even when up to 50% of the coronoid height is involved, as long as CT scans confirm concentric joint alignment and adequate soft-tissue support [1,13]. Radiographic findings such as nonunion or mild incongruity have also shown poor correlation with symptoms or function, supporting a more functional and stability-focused approach rather than a purely morphology-based algorithm [1,13].

In this context, the present findings are compatible with selective fixation algorithms that prioritize intraoperative assessment. The present study was not designed as a validation study of the traditional 50% coronoid height-loss threshold. Accordingly, the findings should not be interpreted as proving or disproving the validity of this cutoff. Rather, because mean height-loss values in both groups were close to this threshold, the present data should be interpreted as evaluating whether coronoid height loss within the observed cohort was associated with functional outcomes, not as validating or invalidating the 50% cutoff. This similarity between the groups follows from the algorithm itself: because fixation was determined by the intraoperative stability test rather than by a size criterion, allocation did not separate patients along the height-loss axis. The absence of a significant association in both univariable and adjusted regression analyses indicates that functional outcome did not vary with coronoid height loss under this algorithm, rather than that fragment size lacks prognostic value in general. Instead, intraoperative fluoroscopic testing of elbow motion after treating the radial head and LUCL appears to be a more practical guide, in line with strategies described in recent studies [14]. These findings are also consistent with the three-column concept, in which the proximal forearm comprises a lateral column formed by the radial head and capitellum, a middle column formed by the anterolateral coronoid facet, and a medial column formed by the anteromedial coronoid facet, with stability depending on the balance between the columns rather than on any single structure [15,16]. Restoration of the lateral column and the lateral ligament complex may therefore be sufficient in selected injuries without direct fixation of a coronoid facet fracture, provided intraoperative testing confirms a concentric reduction.

Because coronoid fixation was performed according to intraoperative stability rather than random allocation, the fixation group may have included elbows with greater perceived instability or complexity at the time of surgery. Therefore, the present comparison should be interpreted as an assessment of a stability-based selective fixation algorithm rather than as a direct comparison of fixation versus non-fixation as independent treatment options. The selective fixation algorithm also requires consistent intraoperative assessment. Because all procedures were performed or directly supervised by a surgeon specializing in elbow surgery, the observed results may reflect not only the algorithm itself but also experience in elbow trauma, fluoroscopic stress assessment, and recognition of subtle residual instability. Therefore, broader application of this approach should consider surgeon experience and the reproducibility of intraoperative stability assessment across centers.

The clinical meaning of these findings is not that coronoid fixation is unnecessary in all terrible triad injuries. Rather, the results support an individualized, stability-based approach in which coronoid fixation is considered together with CT-based morphology and intraoperative dynamic stability after radial head and LUCL reconstruction. Future studies should explore whether combined criteria incorporating CT-based morphology, facet involvement, and dynamic stability assessment may be more reliable than fragment size alone. 

The measurement of coronoid fragment size warrants specific consideration. No validated reference standard for coronoid height measurement is currently available. A recent systematic review of thirty studies identified nineteen distinct measurement techniques, with measurement accuracy formally assessed in only one study and observer reliability reported in fewer than half. Reported values therefore depend substantially on the imaging modality, anatomical landmarks, and measurement plane selected, and absolute percentages obtained by different techniques are not numerically interchangeable. This limitation applies equally to the studies from which the widely cited 50% threshold is derived [17]. For this reason, all measurements in the present study were performed with a single standardized definition, using two lines parallel to the ulnar shaft, one of which was tangent to the base of the trochlear notch, as illustrated by Ahn et al. [3], and applied identically by both observers to every patient. Because no measurement technique can currently be validated against a reference standard, we considered it important to establish whether our findings depended on the particular continuous measurement used. All primary comparisons were therefore repeated using coronoid fracture classification, which was assigned from the same CT studies but is independent of the continuous height-loss measurement. The direction and magnitude of the associations were unchanged, and no significant relationship with functional outcome emerged. This concordance indicates that the absence of an association between coronoid fragment size and functional outcome in this cohort is not an artifact of the measurement technique selected, and that the conclusions of this study do not rest on the continuous height-loss measurement alone.

This study has several limitations. Follow-up was sufficient to characterize recovery of range of motion and patient-reported function but not the later development of post-traumatic osteoarthritis, and radiographic assessment of degenerative change was not part of the outcome set. The findings therefore describe medium-term functional outcomes and cannot exclude a later degenerative divergence between the fixed and non-fixed groups. The retrospective design, relatively small sample sizes, and exclusion of patients requiring secondary procedures from continuous functional outcome analysis limit broader applicability. Because these patients represent treatment failures rather than random missing observations, their exclusion is a potential source of attrition bias. The all-treated sensitivity analysis evaluated unplanned secondary surgery as a separate adverse endpoint across the full cohort; it does not correct the potential bias in the continuous functional outcome comparisons introduced by this exclusion, which therefore remains a limitation. Group allocation depended on intraoperative stability rather than randomization; therefore, confounding by indication cannot be excluded. Patients who underwent coronoid fixation may have represented more unstable or complex injuries at the time of surgery. Accordingly, the comparison between fixation and non-fixation groups should not be interpreted as a randomized comparison of two independent treatment strategies, but rather as an evaluation of outcomes under a selective fixation algorithm. This study was not designed as an equivalence or non-inferiority trial; therefore, the absence of statistically significant differences should not be interpreted as proof of equivalence or as evidence that coronoid fragment size has no clinical relevance. The single-surgeon-supervised setting may have reduced variability in intraoperative decision-making, but it may also limit the generalizability of the findings to surgeons or centers with different levels of elbow trauma experience. Although rehabilitation adherence was reviewed using written checklists and documented in the medical records during follow-up, it was assessed as part of routine clinical care rather than quantified using a validated compliance scale; therefore, rehabilitation compliance could not be included as a separate variable in the statistical adjustment. Despite the adjusted analyses, residual confounding from factors that were not quantified in a standardized manner, including detailed soft-tissue injury severity and rehabilitation-course variability, cannot be excluded. Measurement reproducibility was high in this cohort; however, ICC values reflect the consistency of repeated measurements rather than independent anatomical validation. As discussed above, no reference standard is currently available against which any coronoid height measurement can be validated, and this study was not designed to determine new threshold values for coronoid height loss. Despite these limitations, the study provides useful clinical evidence supporting selective fixation algorithms and underscores the need for future research aimed at refining decision-making in terrible triad injuries.

## 5. Conclusions

In this retrospective cohort of terrible triad injuries treated using a structured selective fixation algorithm, coronoid height loss was not significantly associated with final range of motion or functional outcome scores after radial head reconstruction and LUCL repair. Within this algorithm, and in a cohort managed under the supervision of a single surgeon, intraoperative stability assessment appeared useful in deciding whether coronoid fixation was required. The study does not independently validate this algorithm or establish a treatment threshold, and the results should not be interpreted as proving equivalence between fixation and non-fixation or as demonstrating that fragment size is clinically irrelevant. Larger prospective studies with longer radiographic follow-up are needed to refine stability-based criteria and to clarify how coronoid height loss should be integrated with fracture morphology and intraoperative stability in surgical decision-making.

## Figures and Tables

**Figure 1:**
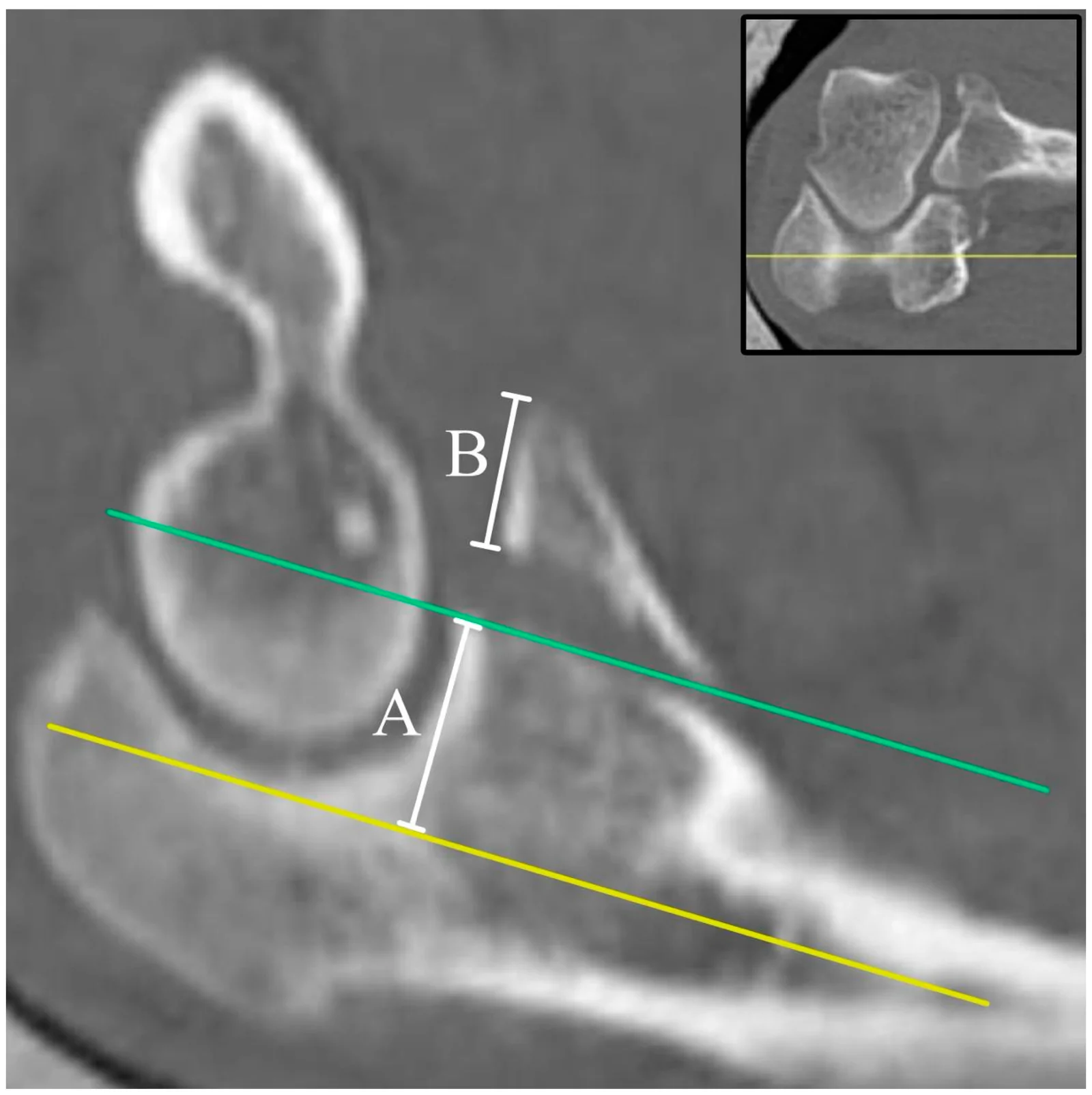
Measurement of coronoid height loss according to the method of Ahn et al. [3]. The mid-sagittal slice was selected with reference to the centre of the proximal ulna on the coronal reconstruction (inset; elbow in 90° of flexion). Two lines parallel to the ulnar shaft are drawn, one through the fracture surface of the residual coronoid process (green) and one tangent to the base of the trochlear notch (yellow). A, residual coronoid height, measured as the perpendicular distance between the two lines; B, height of the displaced fragment, measured on the fragment itself. Coronoid height loss = B/(A + B) × 100.

**Figure 2:**
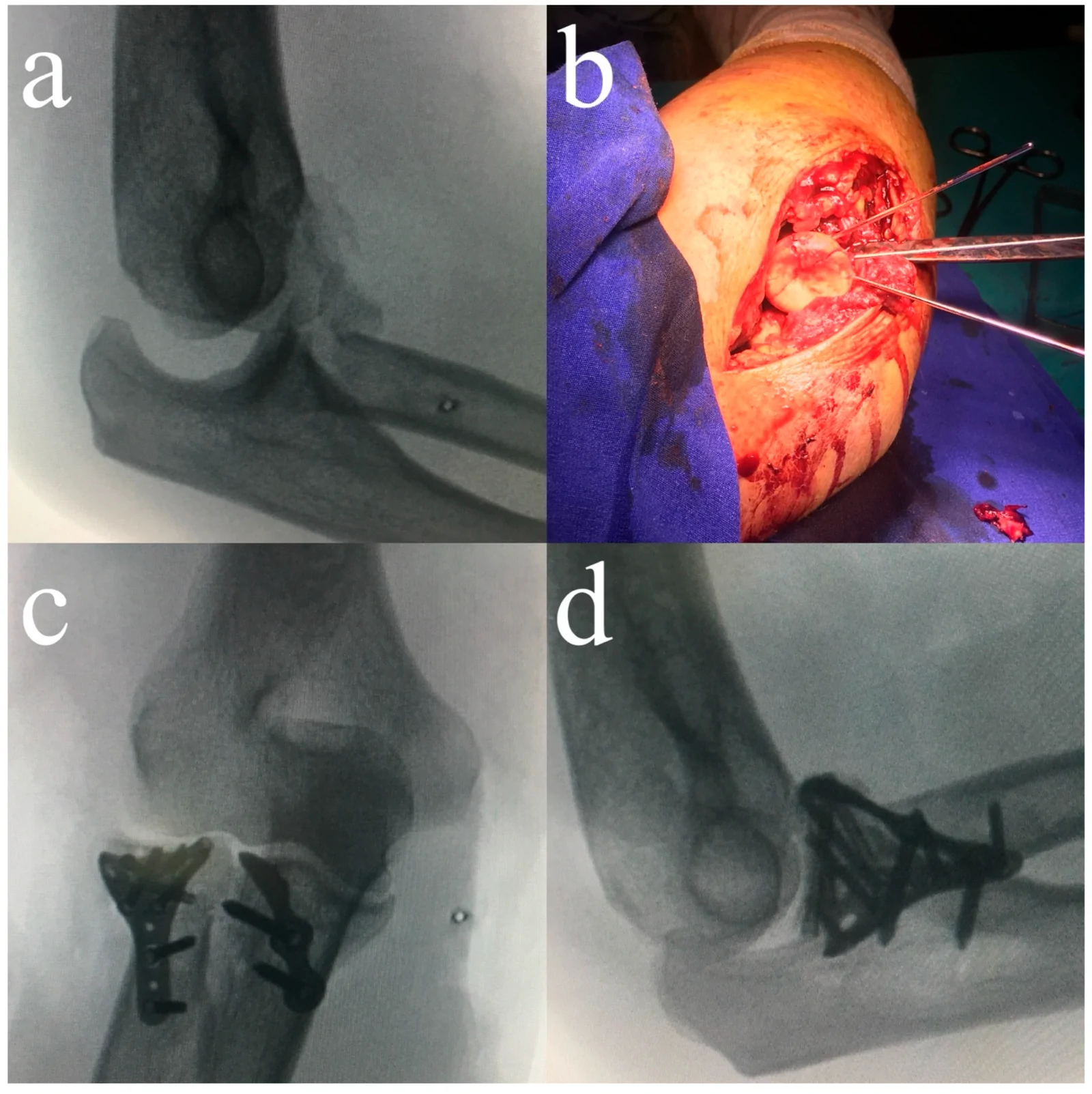
(**a**) Lateral elbow radiograph demonstrating instability accompanied by radial head and coronoid fractures. (**b**) Intraoperative image showing temporary fixation of the radial head using Kirschner wires. (**c**,**d**) Due to persistent instability despite LUCL repair, coronoid fixation was performed. The final anteroposterior (**c**) and final lateral (**d**) fluoroscopic radiographs obtained after coronoid fixation demonstrate restoration of elbow congruency and overall joint stability.

**Table 1:** Patient demographics.

Variable	Group 1 (n = 29)	Group 2 (n = 25)	*p*-Value
Age (years)	42.38 ± 9.87	42.17 ± 9.58	0.94
Sex (M/F)	18/11	15/10	1.00
Dominant side (Dominant/Non-dominant)	17/12	13/12	0.48
Follow-up (months)	27.86 ± 2.85 (24–33)	28.40 ± 2.57 (25–32)	0.40
Time from injury to surgery (hours)	13.34 ± 4.34 (7–21)	12.16 ± 3.92 (8–20)	0.35

**Table 2:** Radial head and coronoid fracture classifications with coronoid height-loss measurements.

Variable	Group 1 (n = 29)	Group 2 (n = 25)	*p*-Value
Mason Type II	14	12	0.92
Mason Type III	15	13	0.93
Morrey Type I	11	12	0.5
Morrey Type II	18	13	0.48
O’Driscoll-tip	11	12	0.51
O’Driscoll Type-anteromedial	18	13	0.49
Height loss (%), mean ± SD	50.21 ± 1.89	49.04 ± 2.74	0.09
Height loss (%), median (IQR)	50.3 (48.9–51.5)	49.1 (47.2–50.8)	
Height loss (%), range (min-max)	46.4–53.9	43.8–54.2	

IQR, interquartile range.

**Table 3:** Comparison of postoperative range of motion and patient-reported outcomes between fixation and non-fixation groups.

Variable	Group 1 (n = 26)	Group 2 (n = 23)	Mean Difference (95% CI)	*p*-Value	Holm-Adjusted *p*-Value
Flex–Ext arc (°)	126.08 ± 12.68	124.74 ± 12.03	1.34 (–5.77 to 8.45)	0.71	1.00
Pro–Sup arc (°)	138.58 ± 30.66	135.57 ± 31.29	3.01 (–14.85 to 20.87)	0.83	1.00
MEPS	87.12 ± 14.01	83.70 ± 14.00	3.42 (–4.65 to 11.49)	0.35	1.00
QuickDASH	22.73 ± 10.23	26.89 ± 10.62	–4.16 (–10.18 to 1.85)	0.14	0.55

Values are presented as mean ± standard deviation. Mean differences are presented as Group 1 minus Group 2. MEPS, Mayo Elbow Performance Score; QuickDASH, Quick Disabilities of the Arm, Shoulder and Hand.

**Table 4:** Univariable and adjusted linear regression analyses evaluating the association between coronoid height loss and functional outcomes.

Outcome	Model	β Coefficient	95% CI	*p*-Value	Holm-Adjusted *p*-Value
Flex–Ext arc	Univariable	–0.16	–0.92 to 0.61	0.68	1.00
Flex–Ext arc	Adjusted	–0.16	–0.99 to 0.67	0.70	1.00
Pro–Sup arc	Univariable	–0.87	–2.76 to 1.02	0.36	1.00
Pro–Sup arc	Adjusted	–0.80	–2.69 to 1.09	0.40	1.00
MEPS	Univariable	0.14	–0.73 to 1.01	0.75	1.00
MEPS	Adjusted	0.12	–0.81 to 1.06	0.79	1.00
QuickDASH	Univariable	–0.31	–0.96 to 0.34	0.34	1.00
QuickDASH	Adjusted	–0.36	–1.04 to 0.32	0.29	1.00

β coefficients represent the change in each outcome per 1% increase in coronoid height loss. Adjusted models included age, sex, Mason type, radial head arthroplasty, and Regan–Morrey coronoid type as covariates. MEPS, Mayo Elbow Performance Score; QuickDASH, Quick Disabilities of the Arm, Shoulder and Hand.

**Table 5:** Functional outcomes according to coronoid fracture classification.

Outcome	Type I (n = 21)	Type II (n = 28)	*p*	Univariable β (95% CI)	*p*	Adjusted β (95% CI)	*p*
Flex-Ext arc	125.1 ± 12.6	125.7 ± 12.2	0.88	0.54 (−6.66 to 7.73)	0.88	1.00 (−7.81 to 9.80)	0.82
Pro–Sup arc	145.1 ± 29.9	131.2 ± 30.4	0.10	−13.96 (−31.49 to 3.56)	0.12	−3.73 (−23.84 to 16.39)	0.71
MEPS	86.7 ± 13.5	84.6 ± 14.5	0.79	−2.02 (−10.20 to 6.15)	0.62	0.63 (−9.23 to 10.50)	0.90
QuickDASH	22.9 ± 10.4	26.1 ± 10.6	0.20	3.18 (−2.92 to 9.29)	0.30	0.36 (−6.94 to 7.65)	0.92

Values are presented as mean ± standard deviation. Regan–Morrey and O’Driscoll classifications were fully concordant in this cohort; type I denotes Regan–Morrey type I/O’Driscoll type 1 (tip) fractures and type II denotes Regan–Morrey type II/O’Driscoll type 2 (anteromedial facet) fractures. β coefficients represent the difference in each outcome for type II relative to type I fractures. Adjusted models included age, sex, Mason type, and radial head arthroplasty as covariates. All Holm-adjusted *p*-values were >0.05. MEPS, Mayo Elbow Performance Score; QuickDASH, Quick Disabilities of the Arm, Shoulder and Hand.

## Data Availability

The raw data supporting the conclusions of this article will be made available by the authors on request.

## Abbreviations

The following abbreviations are used in this manuscript:

LUCL	Lateral ulnar collateral ligament
CT	Computed tomography
ICC	Intraclass correlation coefficients
MEPS	Mayo Elbow Performance Score
QuickDASH	Quick Disabilities of the Arm, Shoulder and Hand
AVN	Avascular necrosis
